# Placebo administration for dry eye disease: a level I evidence based systematic review and meta-analysis

**DOI:** 10.1007/s11096-022-01439-y

**Published:** 2022-08-08

**Authors:** Julia Prinz, Nicola Maffulli, Matthias Fuest, Peter Walter, Frank Hildebrand, Filippo Migliorini

**Affiliations:** 1 RWTH Aachen University Hospital, Pauwelsstraße 30, 52074 Aachen, Germany; 2 Department of Medicine, Surgery and Dentistry, University of Salerno, 84081 Baronissi, SA Italy; 3 Queen Mary University of London, Barts and the London School of Medicine and Dentistry, Mile End Hospital, London, E1 4DG England; 4 School of Pharmacy and Bioengineering, Keele University Faculty of Medicine, Stoke on Trent, England

**Keywords:** Dry eye disease, Keratoconjunctivitis sicca, Placebo, Xerophthalmus

## Abstract

**Background:**

The efficacy of various common treatment options for dry eye disease (DED) has been investigated against placebo. However, the potential beneficial effect of placebo in the management of DED is still unclear.

**Aim:**

This meta-analysis investigated the impact of placebo administration in DED in Ocular Surface Disease Index (OSDI), Schirmer I test (SIT), tear breakup time (TBUT), corneal staining, and complications.

**Method:**

This meta-analysis and systematic review was conducted according to the 2020 PRISMA guidelines. In March 2022, Pubmed, Web of Science, Google Scholar, and Embase were accessed. All the randomised clinical trials which investigated any active treatment against a placebo control group were considered. The following data were extracted at baseline and at last follow-up: Ocular Surface Disease Index (OSDI), tear breakup time test (TBUT), Schirmer I test (SIT), corneal staining.

**Results:**

Data from 56 studies (12,205 patients) were retrieved. Placebo administration is not effective in improving TBUT (*P* = 0.3), OSDI (*P* = 0.2), SIT (*P* = 0.1) and corneal staining (*P* = 0.1) from baseline to last follow-up. Active treatment led to a higher TBUT and SIT compared to placebo administration (*P* < 0.0001). The active treatment resulted in a lower OSDI compared to placebo administration (*P* = 0.0005). Five studies reported data on the corneal staining. No difference was found between placebo administration and active treatment (*P* = 0.8).

**Conclusion:**

Placebo administration does not impact symptoms of DED and can be successfully employed to evaluate the efficacy of active treatments.

**Supplementary Information:**

The online version contains supplementary material available at 10.1007/s11096-022-01439-y.

## Impact statements

- Placebo administration is not effective in improving symptoms of dry eye disease
- Placebo administration can be considered as a safe passive comparator to evaluate efficacy and safety of active treatments in dry eye disease

## Introduction

Dry eye disease (DED) is a common condition of the ocular surface [1], with a prevalence of up to 50% of the global population [2]. Etiological factors include ocular surface inflammation and damage, neurosensory abnormalities, and tear film instability, which is caused by insufficient tear production or quality of the tear film [3]. The main subtypes of DED are aqueous deficient and evaporative DED, with frequent co-existence of both subtypes [2]. DED results in visual disturbance, burning, pain, and photophobia [4, 5]. Conventional therapies for DED include artificial tears, punctal occlusion, topical corticosteroids or secretagogues, and oral essential fatty acid supplementation [6–9]. Artificial tears are a mainstay therapy as they provide an affordable and immediate relief [10]. However, as inflammation is a key component in the pathogenesis of DED, artificial tears might be inadequate in improving the ocular surface damage in patients with more severe DED. Recently, the importance of drugs with anti-inflammatory or secretagogue properties has been highlighted in such cases [11].

In randomized controlled trials (RCTs), the efficacy of an active treatment is commonly evaluated by the difference in outcome between the intervention and placebo group [12]. The placebo effect describes a phenomenon of improvement of symptoms in patients receiving an inert substance [13]. Placebo administration can actually be more beneficial than no-treatment in many clinical settings [14]. Therefore, the clinical impact of a placebo might be neglected [15], resulting in an “efficacy paradox” [16]. This term describes the discrepancy between the treatment efficacy suggested by RCTs and the treatment efficacy observed in the clinical practice [16]. The rate of placebo effect in RCTs investigating various conditions is estimated at 30–40% [17–19]. The placebo effect lowers the statistical power of RCTs, challenging the interpretation of the treatment effects [20]. Placebo effects have been attributed to complex processes, such as behavioural conditioning, patients’ expectations, regression to the mean, and the *Hawthorne* effect [21–24]. Regression to the mean describes that extreme outliers tend to a more average value [24]. In clinical practice, patients with a symptomatic condition often tend to improve spontaneously, even without treatment [24]. The *Hawthorne* effect relates to a change in behaviour of individuals in response to the perception of being observed [25]. Therefore, it affects the generalisability of RCTs to clinical practice [25]. Placebo administration is a major methodological challenge in RCTs investigating DED [27]. The special feature of DED trials is that placebo administration might involve adding a liquid to the ocular surface with possible inherent therapeutic effects in DED [27]. Recently, the processes involved in inducing a placebo effect have been investigated, but the exact mechanisms underlying the placebo effects are not fully understood yet [26]. In some RCTs, patients received a placebo prior to the randomisation procedure to reduce the placebo effects during the treatment period [9, 28, 29]. However, placebo effects were evidenced also in these studies [27]. The efficacy of various common treatment options for DED has been investigated in the clinical setting using placebo as a comparator [7, 9, 29–85].

## Aim

This meta-analysis investigated the impact of placebo administration in DED in Ocular Surface Disease Index (OSDI), Schirmer I test (SIT), tear breakup time (TBUT), corneal staining, and complications.

## Method

### Eligibility criteria

All the randomised clinical trials which investigated any active treatment with a placebo control group were accessed. According to the authors language capabilities, articles in English, German, Italian, French and Spanish were eligible. Only level I of evidence studies, according to Oxford Centre of Evidence-Based Medicine [86], were considered. Only studies published in peer reviewed journals were considered. Reviews, opinions, letters, editorials were not considered. Animal and in vitro studies were not eligible.

### Search strategy

This meta-analysis and systematic review was conducted according to the Preferred Reporting Items for Systematic Reviews and Meta-Analyses: the 2020 PRISMA statement [87]. The PICOT algorithm was preliminary pointed out:

- P (Population): DED;
- I (Intervention): Placebo;
- C (Comparison): Different active treatment (including artificial tears, omega-3 and omega-6 fatty acids, intense pulsed light, acupuncture, cyclosporine, loteprednol, betamethasone, rebamipide, diquafosol tetrasodium, uridine, lifitegrast, botulinumtoxin-A, CF101 (adenosine receptor agonist), SkQ1 (Visomitin), SAR 1118 (integrin antagonist), isunakinra (topical interleukin-1 receptor inhibitor), bevacizumab, canakinumab, secukinumab, Royal Jelly, vitamin A, D-3-Hydroxybutyrate, tretinoin, olopatadine hydrochloride, OTX-101, thymosin b4)
- O (Outcomes): Ocular Surface Disease Index; Tear breakup time test; Schirmer I test, Corneal Staining.

In March 2022, the following databases were accessed: PubMed, Web of Science, Google Scholar, Embase. Only randomized controlled trials were taken into consideration. No time constraint was set for the search. The following keywords were used for the search bar with the Boolean operators AND/OR: *xerophthalmus, dry eye disease, xeropthalmia, placebo, management, therapy, Ocular Surface Disease Index; Tear breakup time test; TBUT; Schirmer I test, SIT; Corneal Staining* (Supplementary material 1).

### Selection and data collection

Two authors (F.M. and J.P.) independently performed the database search. All the resulting titles were screened and, if suitable, the abstract was accessed. The full-texts of the abstracts which matched the topic of interest were accessed. If the full-text of the article was not retrievable or accessible, the study was excluded. The bibliography of the full-text articles was also screened by hand. Any disagreements were discussed and settled by consensus.

### Data items

Two authors (F.M. and J.P.) independently performed data extraction. Study generalities (author, year, journal, number of patients, mean age, women) were extracted. The following data were extracted at baseline and at last follow-up: OSDI [88], TBUT [89], SIT [90], corneal staining.

### Assessment of the risk of bias and quality of recommendations

The between studies risk of bias assessment was evaluated using the risk of bias tool of the Review Manager software (The Nordic Cochrane Collaboration, Copenhagen). The following biases were evaluated by an author independently (J.P.): selection, performance, detection, attrition, reporting, other sources of bias. To investigate the overall risk of publication bias, the funnel plot of the most reported outcome was performed. To grade the quality (or certainty) of evidence and strength of recommendations, the Grading of Recommendations Assessment, Development and Evaluation (GRADE) was performed [91].

### Synthesis methods

The statistical analysis was performed by one authors (F.M.). To assess the improvement from baseline to the last follow-up, the IBM SPSS software version 25 was used. Mean difference (MD) and unpaired t-test were evaluated. For the comparisons, a meta-analysis was conducted using the Review Manager software (The Nordic Cochrane Collaboration, Copenhagen) version 5.3. Data were analyzed using the inverse variance and MD effect measure. The comparisons were performed with a fixed model effect as set up. Heterogeneity was assessed through the $\chi$^2^ and the Higgins-I2 test. If $\chi$^2^ < 0.05 and I^2^ test > 60%, a random model effect was adopted. The confidence intervals (CI) were set at 95% in all analyses. Values of *P* < 0.05 were considered statistically significant. Forest plots were performed for each comparison.

## Results

### Study selection

**Fig. 1:**
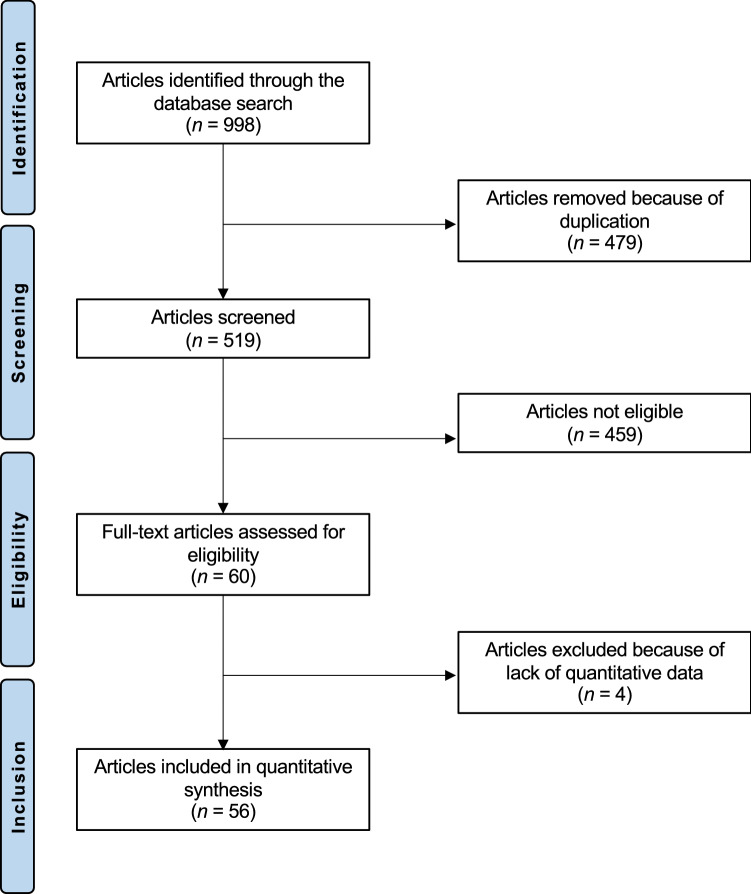
Flow chart of the literature search

After screening the resulting titles, 998 articles were accessed. Of them, 479 were duplicates. A further 459 articles were excluded because of lack of randomization (n = 106), not comparing directly placebo with an active treatment (n = 211), not focusing on the topic (N = 44), language incompatibility (N = 7), including patients with Sjögren’s syndrome (N = 36), including postmenopausal patients (N = 16) or improper study design (N = 40). A further four studies were excluded as they lacked quantitative data. Finally, 56 articles were included in the meta-analysis. As a placebo, the studies included in this meta-analysis used saline [30, 33, 34, 37, 45, 47, 48, 53, 57, 71, 72, 74, 77] or balanced salt solution [35], vehicle drops [29, 43, 50, 52, 56, 61, 66–68, 80], artificial tears [81], sham pulsed light treatment [55, 78], sham acupuncture [92], an ophthalmic solution containing base only [36], oral vitamin E [39], olive oil [7, 42, 44, 51, 70, 75, 83], sunflower oil [59, 85], safflower oil [84], wheat germ oil [65], corn oil [54], palm and coconut oil [62, 63], medium-chain fatty acids [60], or tablets without the active ingredient with the same appearance as the active treatment [40, 46, 49, 58, 69, 79], placebo beverage with a similar texture, flavour, and taste as the active agent [31],1000 IU of vitamin A in a study using 100,000 IU of vitamin A as active treatment [76], or the placebo was not exactly specified [9, 41, 73, 82]. Therefore, the choice of placebo was heterogenous. The search strategy used for literature search in PubMed is reported in Supplementary material 1. The literature search results are shown in Fig. 1.

### Study risk of bias assessment

**Fig. 2:**
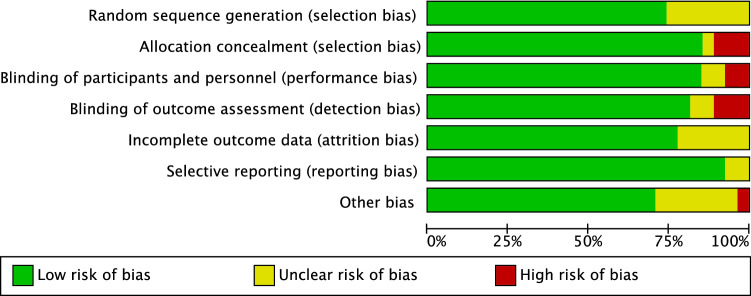
Methodological quality assessment

Given the randomised nature of the studies selected, the risk of selection bias was low. Most authors performed blinding of participants, personnel, and assessor, leading to an overall low risk of performance and detection biases. Equally, the risk of detection and attrition biases were low. Finally, the risk of other bias was low to moderate. Concluding, the risk of bias graph evidenced a good quality of the methodological assessment (Fig. 2).

### Risk of publication bias

**Fig. 3:**
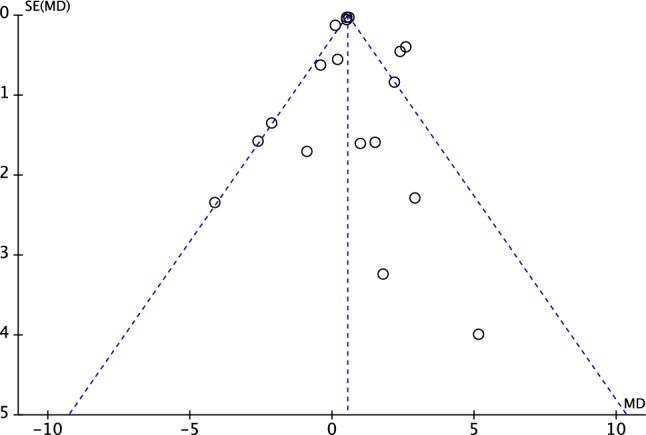
Funnel plot

To assess the overall risk of publication bias, the funnel plot of the most commonly reported outcome was performed and evaluated (SIT). The plot evidenced good symmetry, and the effects were located withing the pyramidal shapes of acceptability (Fig. 3). This indicates a low risk of publication bias.

### Study characteristics and results of individual studies

Data from 4934 patients were retrieved. 73% (3602 of 4934 patients) were women. The mean follow-up was 12.2 ± 16.0 weeks. The mean age was 50.3 ± 14.5 years. Baseline comparability between the placebo and active treatment was found in terms of TBUT (*P* = 0.8), OSDI (*P* = 0.7), SIT (*P* = 0.7), corneal staining (*P* = 0.7). Generalities of the included studies are reported in Table 1, and the analysis of the baseline comparability is shown in Supplementary material 2.

**Table 1:** Generalities and patient baseline of the included studies

Author, year	Journal	Treatment	Patients (*n*)	Mean age	Women (%)
Aragona et al. 2002 [74]	*Br J Ophthalmol*	Sodium hyaluronate	19	50.2	79
		Placebo: saline	25	50.7	80
Asbell et al. 2018 [75]	*N Engl J Med*	Omega–3 eicosapentaenoic and docosahexaenoic fatty acids	349	58.3	81
		Placebo: olive oil	186	57.5	81
Avni et al. 2010 [68]	*Ophthalmology*	CF101 (adenosine receptor agonist)	35		73
		Placebo: vehicle-filled pills	33		56
Baek et al. 2016 [48]	*Curr Eye Res*	Diquafosol tetrasodium, additionally standard postoperative care including prednisolone acetate and moxifloxacin	32	67.7	72
		Placebo: saline, additionally standard postoperative care including prednisolone acetate and moxifloxacin	32	67.7	72
Bhargava et al. 2015 [54]	*Cornea*	Omega-3 eicosapentaenoic and docosahexaenoic fatty acids	240		100
		Placebo: corn oil	256		100
Bhargava et al. 2016 [44]	*Eye Contact Lens*	Omega-3 eicosapentaenoic and docosahexaenoic fatty acids	256	28.9	
		Placebo: olive oil	266	29.6	
Bhargava et al. 2016b [51]	*Curr Eye Res*	Omega-3 eicosapentacenoic and docosahexaenoic fatty acids	65	47.7	62
		Placebo: olive oil	65	48.9	58
Brzheskiy et al. 2015 [52]	*Adv Ther*	SkQ1 (Visomitin)	120	47.5	79
		Placebo: vehicle (benzalkonium chloride, hypromellose, sodium chloride, sodium dihydrogen phosphate dihydrate, and sodium dihydrogen phosphate dodecahydrate)	120	46.3	79
Chang et al. 2009 [79]	*J Korean Med Sci*	Uridine	15	55.0	89
		Placebo: vehicle (L-glutamine, lactose and crystalline cellulose)	12	55.0	89
Chinnery et al. 2017 [42]	*Ophthalmic Physiol Opt*	Omega-3 eicosapentacenoic and docosahexaenoic fatty acids	8	42.0	75
		Placebo: olive oil	4	46.0	75
Choi et al. 2019 [33]	*Graefes Arch Clin Exp Ophthalmol*	Botulinumtoxin-A	26 eyes	60.2	85
		Placebo: Sham injection	26 eyes	55.3	77
Craig et al. 2015 [55]	*Invest Ophthalmol Vis Sci*	Intense pulsed light	28	45.0	71
		Placebo: light therapy with white-blocking filter	28	45.0	71
Deinema et al. 2017 [7]	*Ophthalmology*	Omega–3 eicosapentaenoic and docosahexaenoic fatty acids: fish oil	19	39.4	47
		Omega–3 eicosapentaenoic and docosahexaenoic fatty acids: krill oil	18	42.3	72
		Placebo: olive oil	17	46.2	82
Donnenfeld et al. 2016 [50]	*Cornea*	Lifitegrast	220	61.0	77
		Placebo: vehicle	111	58.8	75
Epitropoulos et al. 2016 [84]	*Cornea*	Omega–3 eicosapentaenoic and docosahexaenoic fatty acids	54	70.4	
		Placebo: safflower oil	51	72.5	
Goldstein et al. 2017 [43]	*Eye Contact Lens*	Isunakinra (topical interleukin-1 receptor inhibitor)	22	73.0	63
		Isunakinra (topical interleukin-1 receptor inhibitor)	22	86.0	65
		Isunakinra (topical interleukin-1 receptor inhibitor)	44	80.0	64
		Placebo: vehicle	30	77.0	59
Goyal et al. 2017 [39]	*Cornea*	Omega–3 eicosapentaenoic and docosahexaenoic fatty acids	30	23.6	55
		Placebo: vitamin E	30	23.6	
Grosskreutz et al. 2015 [53]	*Cornea*	Canakinumab	22	54.0	77
		Secukinumab	25	55.0	72
		Placebo: saline	24	59.0	75
He et al. 2017 [35]	*Medicine (Baltimore)*	Hydroxypropyl methylcellulose	72	68.4	60
		Placebo: balanced salt solution	77	69.4	67
Holland et al. 2017 [41]	*Ophthalmology*	Lifitegrast	354	58.8	76
		Placebo: not specified	357	58.6	76
Hussain et al. 2020 [83]	*Ocul Surf*	Omega–3 eicosapentaenoic and docosahexaenoic fatty acids	22	58.2	86
		Placebo: olive oil	21	58.4	81
Inoue et al. 2017 [40]	*Plos One*	Royal Jelly	22	29.6	29
		Placebo: tablet without the active ingredient with the same appearance as the active treatment	19	37.0	54
Järvinen et al. 2011 [63]	*Cornea*	Sea buckthorn (Hippophae rhamnoides) oil	52	45.0	85
		Placebo: palm and coconut oil triacylglycerols of medium-chain fatty acids	48	46.0	85
Johnson et al. 2006 [72]	*Graefes Arch Clin Exp Ophthalmol*	Sodium hyaluronate	13		62
		Sodium hyaluronate	13		62
		Placebo: saline	13		62
Kangari et al. 2013 [60]	*Ophthalmology*	Omega–3 eicosapentaenoic and docosahexaenoic fatty acids	33	60.6	55
		Placebo: medium-chain triglyceride oil	31	61.8	65
Kasetsuwan et al. 2020 [30]	*Plos One*	Bevacizumab and sodium hyaluronate	19	52.6	89
		Placebo: saline and sodium hyaluronate	12	53.5	83
Katz et al. 1995 [76]	*Invest Ophthalmol Vis Sci*	Vitamin A (100,000 IU)	1871		47
		Placebo: Vitamin A (1000 IU)	1711		48
Kawakita et al. 2013 [58]	*Biomed Res*	Omega–3 eicosapentaenoic and docosahexaenoic fatty acids	15	52.5	67
		Placebo: tablet without the active ingredient with the same appearance as the active treatment	12	51.9	91
Kawakita et al. 2016 [47]	*Sci Rep*	D-3-Hydroxybutyrate	26	59.7	97
		Placebo: saline	31	59.0	97
Kawashima et al. 2019 [31]	*Ocul Surf*	H2-producing milk (prepared by adding galactooligosaccharide, maltitol, and glucomannan to a milk solution comprising cow's milk, and skim milk)	27	42.4	52
		Placebo: beverage with a similar texture, flavour, and taste like the active agent	27	42.5	46
Kaya et al. 2015 [57]	*Acta Ophthalmol*	Hyaluronic acid	16	27.0	50
		Placebo: saline	16	27.0	50
Kinoshita et al. 2012 [9]	*Ophthalmology*	Rebamipide (1%)	103	55.2	90
		Rebamipide (2%)	102	55.2	84
		Placebo: not specified	103	55.2	87
Kokke et al. 2008 [70]	*Cont Lens Anterior Eye*	Omega-6 fatty acid: evening primrose oil	28	46.4	100
		Placebo: olive oil	24	37.3	100
Larmo et al. 2010 [62]	*J Nutr*	Sea buckthorn (Hippophae rhamnoides) oil	52	45.0	85
		Placebo: palm and coconut oil triacylglycerols of medium-chain fatty acids	48	46.0	85
Mah et al. 2008 [71]	*Curr Med Res Opin*	Olopatadine hydrochloride	25	55.5	58
		Placebo: saline	27	55.5	58
Malhotra et al. 2019 [32]	*Cornea*	OTX-101 (0.09%)	487	58.6	84
		OTX-101(0.05%)	142		84
		Placebo: vehicle	505	59.6	84
Olenik et al. 2013 [85]	*Clin Interv Aging*	Omega–3 eicosapentaenoic and docosahexaenoic fatty acids	33	58.0	73
		Placebo: sunflower oil	31	54.0	71
Petrov et al. 2016 [46]	*Adv Ther*	SkQ1 (1.55 µg/mL)	30	62.0	74
		SkQ1 (0.155 µg/mL)	30	62.0	74
		Placebo: vehicle	31	62.0	74
Pflugfelder et al. 2004 [80]	*Am J Ophthalmol*	Loteprednol etabonate	32	57.6	63
		Placebo: vehicle	34	56.2	88
Schmidl et al. 2017 [37]	*J Ocul Pharmacol Ther*	C-NAC (single instillation)	21	36.0	71
		Placebo: saline (single installation)	21	36.0	71
		C-NAC (once daily for 5 days)	17	24.0	77
		C-NAC (twice daily for 5 days)	17	24.0	77
Selek et al. 2000 [73]	*Eur J Ophthalmol*	All-trans-retinoic acid (tretinoin)	22	53.8	
		Placebo: not specified	22		
Semba et al. 2000 [82]	*Am J Clin Nutr*	Vitamin A (healthy subjects)	59	4.9	71
		Placebo: not specified (healthy subjects)	59	4.9	71
		Vitamin A	58	4.9	71
		Placebo: not specified	60	4.9	71
Semba et al. 2012 [61]	*Am J Ophthalmol*	SAR 1118 (Integrin Antagonist, 0.1%)	54	63.1	83
		SAR 1118 (Integrin Antagonist, 1%)	51	63.6	70
		SAR 1118 (Integrin Antagonist, 5%)	48	62.3	81
		Placebo: vehicle	48	60.4	78
Sheppard et al. 2014 [29]	*Ophthalmology*	Lifitegrast	295	61.1	74
		Placebo: vehicle	293	60.2	78
Sheppard Jr et al. 2013 [59]	*Cornea*	Gamma-linolenic acid and omega-3 fatty acids	19	62.0	100
		Placebo: sunflower oil	19	61.0	100
Shin et al. 2010 [92]	*Acta Ophthalmol*	Acupuncture	21	40.5	76
		Placebo: sham acupuncture	21	42.8	71
Shokoohi-Rad et al. 2020 [77]	*Indian J Ophthalmol*	Betamethasone acetate	28	66.0	13
		Placebo: saline	34	64.6	13
Sosne et al. 2015 [56]	*Cornea*	Thymosin b4 (RGN-259)	6	54.2	67
		Placebo: vehicle	3	63.7	67
Szegedi et al. 2018 [34]	*J Ocul Pharmacol Ther*	Sodium hyaluronate, triglycerides, and phospholipids	20	34.6	70
		Sodium hyaluronate	20	40.5	65
		Placebo: saline	20	39.2	80
Toshida et al. 2017 [36]	*Drug Des Devel Ther*	Vitamin A palmitate	66	45.8	91
		Placebo: an ophthalmic solution containing base only	33	52.1	91
Villani et al. 2011 [66]	*Cornea*	T-Clair SPHP700-3	30		
		Placebo: vehicle	27		
Vogel et al. 2010 [67]	*Am J Ophthalmol*	Sodium hyaluronate	217	60.7	78
		Placebo: vehicle	219	62.2	72
Wang et al. 2016 [49]	*Inflammopharmacology*	Omega 3 fatty acids (100%)	60	33.9	43
		Omega 6 fatty acids (100%)	60	34.6	42
		Omega 3, omega 6 (50%, 50%)	60	32.2	46
		Omega 3, omega 6 (75%, 25%)	60	35.3	44
		Omega 3, omega 6 (25%, 75%)	60	34.9	42
		Placebo: tablet without the active ingredient with the same appearance as the active treatment	60	34.2	45
Willen et al. 2008 [81]	*Eye Contact Lens*	Cyclosporine A	22	44.0	1
		Placebo: artificial tears	22	42.2	1
Wojtowicz et al. 2011 [65]	*Cornea*	Omega-3 eicosapentacenoic and docosahexaenoic fatty acids: fish oil	21	61.0	56
		Placebo: wheat germ oil	15	61.0	56
Xue et al. 2020 [78]	*Ocul Surf*	Pulsed light (4 flashes)	28	48.0	68
		Pulsed light (5 flashes)	29	56.0	62
		Placebo: sham treatment	30	55.0	70

### Efficacy of placebo administration

Placebo administration is not effective in improving TBUT (*P* = 0.3), OSDI (*P* = 0.2), SIT (*P* = 0.1), and corneal staining (*P* = 0.1) from baseline to last follow-up (Supplementary material 3).

### Efficacy of placebo administration compared to the active treatment

**Fig. 4:**
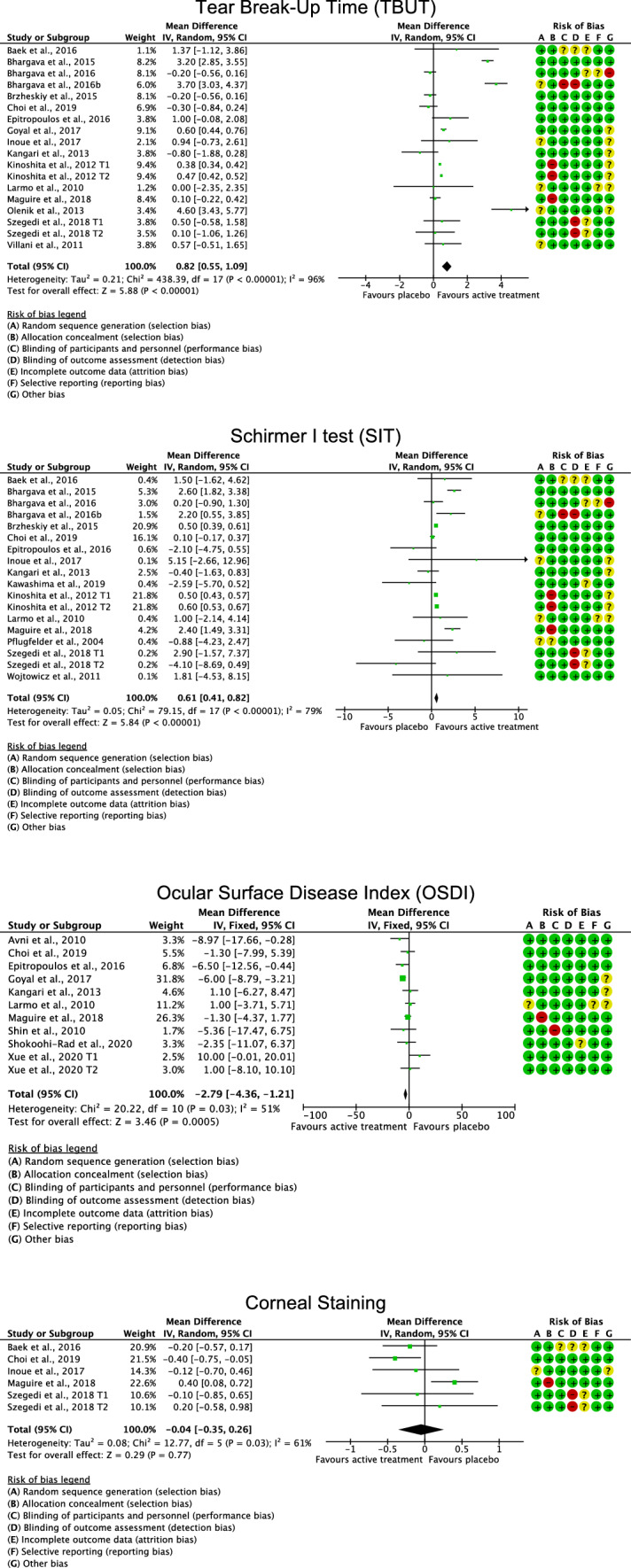
Forest plots

Active treatment evidenced a higher TBUT (MD 0.82; 95% CI 0.55 to 1.09; *P* < 0.0001), SIT (MD 0.61; 95% CI 0.41 to 0.82; *P* < 0.0001), and OSDI (MD − 2.79; 95% CI − 4.26 to − 1.21; *P* = 0.0005) compared to placebo administration. No difference was found between placebo administration and active treatment in corneal staining (*P* = 0.8). These results are shown in greater detail in Fig. 4

### Quality of the recommendations

**Fig. 5:**
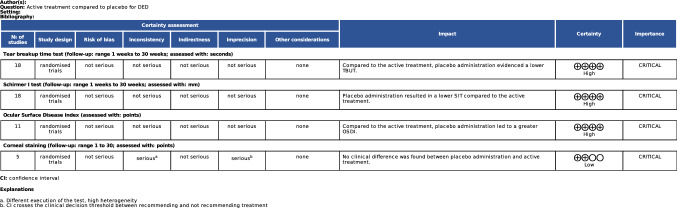
GRADE

The level of evidence quality according to the GRADE system was high for TBUT, SIT, and OSDI, whereas a low level of evidence in quality for the corneal staining was evidenced (Fig. 5).

## Discussion

### Statement of key findings

The present study demonstrated that placebo administration did not improve symptoms of DED at the last follow-up. Placebo administration can therefore be considered as passive comparator to evaluate the efficacy and safety of active treatments in DED.

### Strengths and weaknesses

The literature search was performed in Pubmed, Web of Science, Google Scholar and Embase, as previously recommended to guarantee efficient coverage [115]. Within the selection and data collection, no severe disagreements between the two responsible authors occurred. Therefore, no impact on internal validity must be expected. The present meta-analysis and systematic review was detailed and precise, but has several limitations. Firstly, the active treatment and the placebos protocols showed a high variability. Hypothetically, different effects might be attributable to different placebos. Selek et al. and Goldstein et al. attributed the symptomatic improvement witnessed in the placebo group to a lubricating effect of the vehicle itself on the ocular surface [73]. Shin et al. evaluated the efficacy of acupuncture for DED, and employed a sham acupuncture control group which they referred to as the placebo group [92]. Yet, placebo acupuncture is technically impossible. Accordingly, “sham” describes any control treatment of acupuncture aiming to make the patients believe that they received the real treatment [116, 117]. A superior efficacy of sham acupuncture compared to pharmacological placebo has been suggested [118]. Conversely, patients demonstrating a therapeutic effect to placebo were excluded after a 2-week-run-in period. Thereby, the risk of a strong placebo effect should be mitigated [61]. Moreover, the heterogeneous length of follow-up might also limit the reliability of our results. Given the lack of quantitative data available for inclusion, no further subgroups were possible to investigate. Hence, conclusions from the present study must be interpreted with caution.

### Interpretation

Previously, Imanaka et al. investigated predictive factors of the placebo effect in trials for DED, collecting data from 205 patients enrolled in 3 placebo-controlled RCTs. High baseline scores and age affected the placebo responses of the corneal staining score [27]. Ageing is an essential risk factor for DED [27]. The high proportion of females (73%) in the present study agrees with previous publications and has been attributed to the effects of sex steroids including oestrogens, glucocorticoids, and epigenetics [93]. While the exact association of sex steroids and DED is still unclear, relatively low levels of serum androgen in females are associated to lower anti-inflammatory effects on the ocular surface, promoting DED [94]. Hypothetically, the placebo might have a beneficial effect in patients with high baseline scores by acting as artificial tears [27]. Also, the greater placebo effect in patients with high baseline scores can be attributed to the regression to the mean phenomenon [24]. In other conditions, such as low back pain, a previous meta-analysis showed rapid symptom improvement in the first 6 weeks and less marked improvement thereafter both in the treatment and placebo groups [95]. Therefore, regression to the mean might commonly be falsely interpreted as efficacy, but occurs simply by chance and with time [96]. Placebo effects have been reported in patients with depression, cardiovascular diseases, asthma, and different pain syndromes [97–101]. Notably, a systematic review collecting data from 72 RCTs with 9827 patients with fibromyalgia and 70 RCTs with 10,297 patients with diabetic neuropathy showed a superior effect of placebo administration compared to non-treatment. The placebo group showed a significant reduction of pain and fatigue [102]. It has been presumed that the examination by a doctor and the actual physical motions of taking medications can have therapeutic effects [96]. Placebo effects have been attributed to the release of substances such as endogenous opioids [103], endocannabinoids [104], dopamine [105], oxytocin [106], and vasopressin [107]. These substances show specific effects to the target system [101]. In addition, neuroimaging studies have shown changes similar to those caused by opioids in brain activation patterns induced by placebo [108]. In DED, patients show positive perceptions regarding the effectiveness of their treatments [109]. The improvement anticipated by the patient might be attributable to the measured improvement [110]. Notably, some of the studies included in this meta-analysis showed some beneficial effect of placebo on SIT [81, 83], TBUT [31, 75], corneal staining [53, 66, 75] or OSDI [7, 75, 79]. The small, nonstatistical reduction of corneal staining induced by placebo may well be regression to the mean [53]. Chang et al. investigated the efficacy of oral uridine compared to placebo on DED. They reported a reduction of corneal staining and improvement of SIT in the uridine compared to the placebo group with statistically significant differences between the groups. Moreover, treatment with uridine significantly reduced the OSDI score. However, the placebo group also showed some reduction of the OSDI score. The authors assume that these nonsignificant differences between the groups are attributable to the placebo effect [79]. In 2018, the Dry Eye Assessment and Management (DREAM) trial concluded that omega-3 fatty acid supplements do not provide better outcomes than placebo in the management of DED [75, 111]. As to adverse events, placebo proved safe in the studies considered for our meta-analysis. However, Kinoshita et al. reported that eye irritation occurred more frequently in the placebo than in the treatment (rebamipide) group. However, the authors did not specify what the placebo consisted of [9]. In the DREAM trial, the percentage of patients with at least one serious adverse event was 8.1% in the placebo group receiving olive oil. The percentage of patients with at least one nonserious adverse event was similar in the active treatment group and the placebo group (61.9% and 60.8%, respectively) [75]. In the DREAM extension study, one patient in the placebo group was hospitalized for dyspnoea as a serious adverse event [83]. A randomized study published in 2017 reported that patients were more likely to report adverse events when they were aware that they received statin therapy than when they were blinded [112]. Therefore, negative expectations of the patients regarding the treatment with either active or inert substance might cause a negative placebo effect, called the nocebo effect [112]. Nocebo effects have been described in many clinical contexts. Up to 19% of adult and 26% of elderly patients receiving placebos report side effects [113]. They have been partly attributed to the verbal suggesting in the context of informed-consent process [101]: in the clinical setting, expectancies might be affected by the preceding description of the treatment [101]. On a neurobiological level, the nocebo effect has been shown to be mediated by cholecystokinin [114] and to be associated with hyperactivity of the hypothalamic–pituitary–adrenal axis [101]. This increased activity is antagonized by benzodiazepine, which suggests the role of anxiety of patients in nocebo effects [101].

### Further research

Altogether, distinguishing whether an observed effect is secondary to placebo, regression to the mean, or simply part of the course of the disease, can be challenging [96]. The exact intensity of the placebo effects in clinical trials on DED remains difficult to determine. In patients with DED, placebo did not show beneficial therapeutic effects, but was safe. However, no subgroup analysis could be performed given the lack of quantitative data available for inclusion. Therefore, further research is warranted to focus on evaluating different types of placebo administration in DED.

## Conclusion

Placebo administration does not impact symptoms of DED and can be successfully administered to evaluate the efficacy of active treatments. These conclusions must be interpreted within the limitations of the present study.

## Supplementary Information

Below is the link to the electronic supplementary material.

Supplementary file 1 (DOCX 17 KB)
