# Supplementary material for: Placebo administration for dry eye disease: a level I evidence based systematic review and meta-analysis
Source: Int J Clin Pharm. 2022 Aug 8;44(5):1087–101. doi: 10.1007/s11096-022-01439-y (PMC9618542; doi:10.1007/s11096-022-01439-y)
Supplement: Supplementary file 1 — (DOCX 17 KB) [file 11096_2022_1439_MOESM1_ESM.docx]

**APPENDIX**

| Database: Pubmed | Terms | Number of results |
| --- | --- | --- |
| #1 | “xerophthalmus“ | 64 |
| #2 | “dry eye disease” | 809 |
| #3 | “xerophthalmia” | 64 |
| #4 | 1 OR 2 OR 3 | 816 |
| #5 | “placebo” | 123,284 |
| #6 | “management” | 82,519 |
| #7 | “therapy” | 449,881 |
| #8 | 2 AND 5 | 185 |
| #9 | 2 AND 7 | 732 |
| #10 | “Ocular Surface Disease Index” | 218 |
| #11 | “Tear breakup time test” | 354 |
| #12 | “TBUT” | 149 |
| #13 | 11 OR 12 | 397 |
| #14 | “Schirmer 1 test” | 298 |
| #15 | “SIT” | 1,882 |
| #16 | 14 OR 15 | 2,171 |
| #17 | “Corneal Staining” | 525 |
| #18 | 2 AND 10 OR 11 OR 14 OR 17 | 864 |

**Supplementary material 1.** Search strategy used for literature search in PubMed. All the titles of the resulting articles were screened independently by two authors.

| **Endpoint** | **Placebo (n = 5632)** | **Treatment (n = 6932)** | **MD** | ***P*** |
| --- | --- | --- | --- | --- |
| TBUT | 5.6 ± 3.0 | 5.4 ± 2.9 | 0.2 | 0.8 |
| OSDI | 32.8 ± 16.0 | 34.7 ± 13.8 | -1.8 | 0.7 |
| SIT | 10.4 ± 4.0 | 10.0 ± 4.0 | 0.4 | 0.7 |
| Corneal staining | 2.7 ± 1.3 | 2.9 ± 1.4 | -0.2 | 0.7 |

**Supplementary material 2.** Baseline comparability

| **Endpoint** | **At baseline** | **At last FU** | **MD** | ***P*** |
| --- | --- | --- | --- | --- |
| TBUT | 5.6 ± 3.0 | 6.0 ± 2.9 | 0.4 | 0.3 |
| OSDI | 32.8 ± 16.0 | 24.8 ± 12.1 | -8.1 | 0.2 |
| SIT | 10.4 ± 4.0 | 9.1 ± 4.1 | -1.3 | 0.1 |
| Corneal staining | 2.7 ± 1.3 | 2.5 ± 1.5 | -0.3 | 0.1 |

**Supplementary material 3.** Efficacy of placebo
